# Metabolic programming in dendritic cells tailors immune responses and homeostasis

**DOI:** 10.1038/s41423-021-00753-1

**Published:** 2021-08-19

**Authors:** Sofie Hedlund Møller, Limei Wang, Ping-Chih Ho

**Affiliations:** 1 Department of Fundamental Oncology, University of Lausanne, Lausanne, Switzerland; 2 Ludwig Institute for Cancer Research, University of Lausanne, Epalinges, Switzerland

**Keywords:** Dendritic cells, Immunometabolism, Homeostasis, Tumor immunity, Microbiota, Antigen processing and presentation, Innate immune cells

## Abstract

It is being increasingly acknowledged that immune cells depend on certain metabolic traits to perform their functions and that the extracellular environment can influence cell metabolism and vice versa. Dendritic cell (DC) subsets traffic through highly diverse environments from the bone marrow, where they develop, to the various peripheral tissues, where they differentiate and capture antigens, before they migrate to the lymph node to present antigens and prime T cells. It is plausible that DC subsets modulate their stimulatory abilities in response to unique metabolic programming. The metabolic requirements of DCs are just recently being discovered, and subset- and context-specific metabolic phenotypes in DCs are highly intertwined with DC functions. In this review, we present the current knowledge on the intrinsic and extrinsic determinants of DC metabolism, how they regulate DC function with examples from tumor biology and in interaction with the microbiota, and discuss how this can be applied therapeutically.

## Introduction

Dendritic cells (DCs) are critical initiators of immune responses to infections or cancer and are vital for maintaining tolerance during homeostasis. DCs are the most useful of antigen presenting cells (APCs) in terms of their ability to migrate to lymph nodes and prime naïve T cells. In addition, DCs secrete cytokines and chemokines to drive proper immune responses. Cell metabolism is being recognized as a conserved regulatory circuit that tailors immune cell function and survival. DCs likewise rely on tightly regulated metabolism to launch an immune response or promote tolerance; however, due to the limitations of in vitro models and the scarcity of DCs in accessible tissues, there are gaps in our understanding of DC metabolism that remain to be filled.

### General DC biology

The most common DCs are conventional DCs (cDCs) and plasmacytoid DCs (pDCs). In addition, epidermal-resident Langerhans cells and monocyte-derived DCs (moDCs) arising from circulating monocytes in the blood are considered DC subgroups. cDCs can be subdivided into cDC1s and cDC2s, which are developed through distinct transcriptional programs [1]. Human and murine DC subsets are highly similar in transcription factor dependency and subset development but have unique surface markers. Human cDC1s and cDC2s are distinguished by their surface expression of CD141 and CD1c, respectively. Mouse cDC1s are recognized by the surface expression of XCR1, CLEC9A, and CD8α (lymphoid-resident) and/or CD103 (migratory), whereas CD11b^+^ SIRPα^+^ cDC2s only express CD103 in nonlymphoid tissues [2] (Table 1). Studies on conventional DCs are generally limited to murine DCs since access to human DCs is largely restricted to peripheral blood mononuclear cell (PBMC)-derived DCs. As such, this review is mostly based on findings from murine studies and will specify when reporting findings from humans. cDC1s and cDC2s exhibit different functional properties. cDC1s excel at activating CD8^+^ T cells by presenting exogenous antigen on major histocompatibility complex class I (MHCI) in a process called cross-presentation. cDC2s, on the other hand, predominantly present antigens on MHCII, activating CD4^+^ T cells, and pDCs are specialized secretory cells that produce large amounts of type I interferons (IFN-I) promoting the response to viral infections (Table 1).

**Table 1:** Common DC subsets and their metabolic requirements

	cDC1	cDC2	pDC	moDC
Main function	Antigen cross-presentation to CD8^+^ T cells, IL-12, IL-6 secretion	Direct antigen-presentation to CD4^+^ T cells, IL-6, TNF-α, IL-23 secretion	IFN-I production	Antigen presentation and TNF-α, IL-12, IL-23 secretion
Transcription factors	Batf3, Irf8, Id2	Irf4, Notch2, Klf4	Irf8, E2-2, Irf7	Klf4, Irf8
Surface markers (mouse)	CD11c, MHCII, XCR1, CLEC9A, CD24, DEC205 CD8α (resident), CD103 (migratory)	CD11c, MHCII, SIRPα, CD11b	CD11c^low^, MHCII^low^, PDCA1, Siglec H, B220. CD209, SIRPα,	CD11c, MHCII, CD11b, Ly6C,CD14, CCR2
Surface markers (human)	CD11c^low^, MHCII, XCR1, CLEC9A, CD141, DEC205	CD11c, MHCII, SIRPα, CD1c,	MHCII^low^, CD123, CLEC4C, CD304, CCR2	CD11c, MHCII, CD11b CD14, CD1c, CD64, CD206, CD209, SIRPα, CD1a, CCR2
Development and differentiation	PI3K/Akt and mTOR is required for Flt3L-induced DC development^a,c^ [10] AMPK-KO or FAO-inhibition increase cDC2/cDC1^a,c^ [11] Mst1/Mst2 deficiency impair Flt3L-expansion of splenic cDC1^c^ [12]	ROS-inhibition increases cDC1/cDC2^a^ [11]	PI3K/Akt and mTOR is required for Flt3L-induced DC development^a,c^ [10]	Increased mitochondrial activity and ROS is required for differentiation into moDCs^d^ [17] Upregulated mitochondrial biogenesis genes PGC-1α, NRF-1, TFAM^d^ [18] Constitutive active PI3K/mTOR signaling^d^ [16]
Steady-state	OXPHOS^high^ Glycolysis^high a,c^ [11, 12, 19] Glycogen storage^b^ [167]	OXPHOS^low^ Glycolysis^low a,c^ [11, 12, 19] Glycogen storage^b^ [167]		
Early Activation	PRR stimulation induces TBK1/IKKε/Akt-dependent glycolysis and FAS^a,b^ [19, 20, 22, 23] Glycolysis and FAS are required for DC maturation^,c^ [19, 23] Glycolysis is required for DC migration^b,c^ [21] Glycogenolysis fuels glycolysis^b^ [167]	TLR induced glutaminolysis-driven OXPHOS required for pDC IFN-I and co-stimulatory molecules^e^ [36] IFN-I-induced FAO enhance autocrine IFN-I and pDC function^a^ [28] Stimuli-dependent reliance on glycolysis for IFN-I production^f^ [35] mTOR-dependent RLR-induced IFN-I production^f^ [47]	PRR stimulation induces glycolysis and iNOS expression^b,c^ [23, 24] Glycogenolysis fuels glycolysis^d^ [167] mTOR-dependent PRR activation^d^ [47]	
Mature DC	Glucose-deprivation 8 h after LPS enhance CD8^+^ T cell priming^b^ [44] Mst1/2-dependent IL-12 production and CD8+ T cell priming^,c^ [12] Lipid bodies promote antigen cross presentation^a^ [79, 80] Lipid overload diminishes antigen uptake^d, f^ [32, 33] NOX2-produced ROS required for antigen processing and CD8^+^ T cell priming^b, c^ [82, 83]	*Contrasting data* mTORC1 blocks MHCII expression and antigen uptake^b^ [42, 69, 70] mTORC1 facilitates MHCII peptide loading ^b, d^ [71, 72]		Glycolysis^high^ and iNOS required for survival^c^ [24] mTOR required for antigen uptake^d^ [45]

^a^In vitro in Flt3L-differentiated BMDCs

^b^In vitro in GM-DCs

^c^Ex vivo/in vivo splenic/lymphoid DCs

^d^In vitro human GM-CSF+IL-4 differentiated moDCs

^e^In vivo human circulating pDCs

^f^Ex vivo/in vivo tumor-infiltrating DCs (TIDCs)

### Metabolic changes and requirements during DC development

**Fig. 1:**
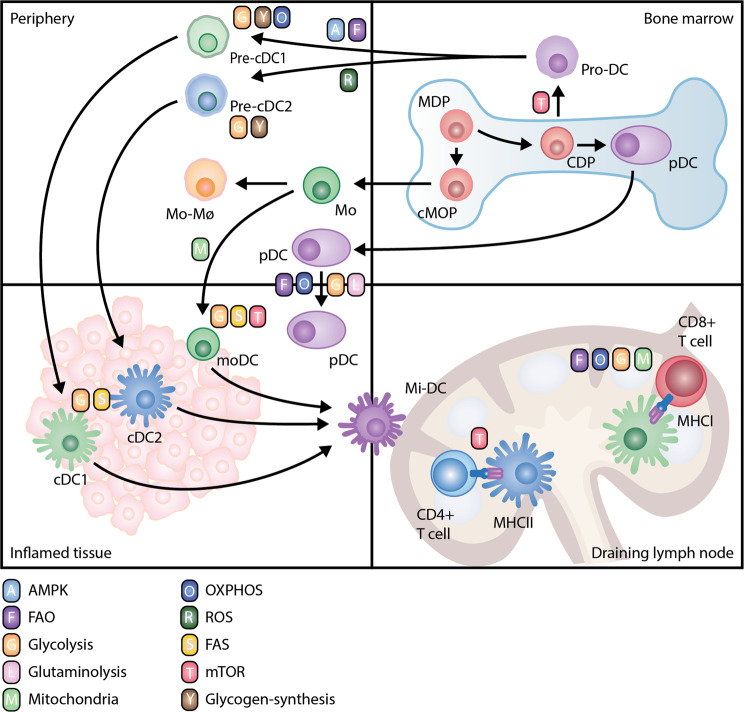
Metabolic reprogramming at early DC activation. Peripheral lymphoid tissue-resident DCs are ultimately of bone marrow (BM) origin via a common restricted precursor termed common dendritic monocyte precursors (CDPs), which further differentiate into DC progenitors (Pro-DCs) and pDCs. Pro-DCs become immature pre-cDC1s and pre-cDC2s with distinct metabolic programming. AMP-activated kinase (AMPK) (A) or fatty acid oxidation (FAO) (F) promote pre-cDC1 differentiation, and reactive oxygen species (ROS) (R) skew DC differentiation toward pre-cDC2. In the periphery, pre-DCs become immature DCs that produce glycogen through glycogen synthesis (Y). Here, cDC1s rely on more OXPHOS (O) and glycolysis (G) than cDC2s. Glycolysis is highly induced under DC activation and maturation, which is supported by glycogen storage and fuels fatty acid synthesis (S). During the formation of mature immunogenic DCs, the DC antigen presentation process is affected by mTOR (T), while cDC1s support their cross-presentation function through the establishment of distinct and tightly regulated metabolic processes, including glycolysis, oxidative phosphorylation (O), mitochondria (M) and fatty acid metabolism. Mo-DC, monocyte-derived DC; Mi-DC, migration DC

cDCs arise from hematopoietic stem cell-derived common myeloid progenitors (CMPs) that develop into common macrophages and DC progenitors (MDPs). MDPs give rise to restricted DC progenitors (CDPs) and common monocyte precursors (cMOPs) in the bone marrow. cMOPs differentiate into monocytes, which in tissues can generate DCs under certain conditions, including in cancer. CDPs can commit to either pre-cDC1 or pre-cDC2 that enter the circulation. Pre-DCs become immature DCs in the periphery, where they subsequently mature after activation by inflammatory stimuli (Fig. 1). pDCs can develop from both CDPs and common lymphoid progenitor cells [3]. However, by using single-cell technologies, researchers have recently revealed that lymphoid and myeloid-derived pDCs have distinct functional and transcriptional profiles despite similar phenotypic markers [4, 5]. Hence, our current categorization of DC subsets might be too simplistic since six different DC clusters and novel DC subsets have been shown in human blood [6] and nonsmall cell lung carcinomas [7, 8], respectively.

Commitment to CMP induces Fms-like tyrosine kinase 3 (Flt3) on the cell surface, which drives DC development. Flt3 ligand (Flt3L) is required for homeostatic DC development, evident from the profound reduction of DCs in Flt3L-deficient mice [9]. Moreover, Flt3L treatment can expand the DC population, which is exploited for in vitro studies and is proposed to have therapeutic prospects, as discussed below. Flt3L-supplemented bone marrow cultures have revealed that Flt3L drives DC development by increasing anabolism, proliferation and differentiation in a phosphoinositide 3-kinase (PI3K)- and mammalian target of rapamycin (mTOR)-dependent manner [10, 11]. Pre-cDC1s and pre-cDC2s become immature cDC1s and cDC2s with distinct transcriptional profiles, but until recently, little was known about the metabolic differences of cDC1s and cDC2s. Flt3L-derived cDC1s have higher mitochondrial content and membrane potential than cDC2s [11], and analysis of splenic CD8α^+^ cDC1s showed that they have higher oxidative phosphorylation (OXPHOS) than their CD8α^−^ counterparts [12], underscoring that cDC1s and cDC2s are metabolically different even when developed in vivo. Interestingly, inhibition of AMP-activated kinase (AMPK) or fatty acid oxidation (FAO) during Flt3L-induced differentiation of bone marrow-derived DCs (BMDCs) has been shown to promote cDC2 differentiation, whereas inhibition of reactive oxygen species (ROS) skewed DC differentiation toward cDC1s [11]. In agreement with this, mice with a deficiency of the AMPK activating kinase liver kinase B1 (LKB1) in CD11c^+^ cells had an increased number of cDC2s versus cDC1s in the thymus [13]. Together, these results suggest that cDC1 differentiation or survival depends on LKB1/AMPK/FAO, whereas cDC2s depend on ROS signaling (Table 1) (Fig. 1). In addition to Flt3L-induced DC differentiation, multiple studies have used granulocyte macrophage-colony stimulating factor (GM-CSF) as a method to generate BMDCs (GM-DCs) or PBMC-derived monocytes cultured with IL-4 (moDCs). Given that GM-CSF promotes DC differentiation from monocytes, it is likely that GM-DCs resemble inflammatory DCs as opposed to homeostatic immature DCs [14]. Xu et al. compared these two culture models, which displayed several striking differences [15]. GM/IL-4-DC cultures do not contain any pDCs and produce more inflammatory mediators than FL-DCs. FL-DCs migrate more efficiently to lymphoid organs. Furthermore, it has been established that human GM-CSF and IL-4-differentiated moDCs are metabolically distinct from circulating myeloid DCs [16]. Metabolically, human moDCs increase the master regulator of mitochondrial biogenesis, peroxisome proliferator-activated receptor gamma (PPARγ) coactivator 1-alpha (PGC-1α), shortly after differentiation induction with GM-CSF and IL-4, and their mitochondrial mass increases during differentiation [17, 18].

### Metabolic reprogramming facilitates dendritic cell activation and maturation

Immature DCs are localized in the circulation or peripheral tissue and become activated by signals elicited upon engagement of pattern recognition receptors (PRRs), including Toll-like receptors (TLRs), retinoic acid-inducible gene I-like receptors (RLRs), nucleotide-binding oligomerization domain-like receptors, and C-type lectin receptors (CLRs). These PRRs allow DCs to respond rapidly to pathogen-associated molecular patterns or danger-associated molecular patterns. DCs continuously take up antigens, which under homeostatic conditions are harmless antigens that should be tolerated. When DCs are activated through PRR stimulation, they increase the expression of chemokine receptors, such as C-C chemokine receptor type 7 (CCR7), which facilitates DC migration to the draining lymph node. Furthermore, PRR activation leads to increased expression of MHC molecules, costimulatory surface molecules (CD40, CD80, CD86), and cytokines (IL-12, IL-10, IL-23, TNF-α), which prompts the transition from resting immature DCs to functional mature DCs capable of activating T cells in the lymph node. Here, we describe the known metabolic reprogramming that occurs in response to DC activation and is required for DC maturation.

#### Glycolytic induction in early and late DC activation

**Fig. 2:**
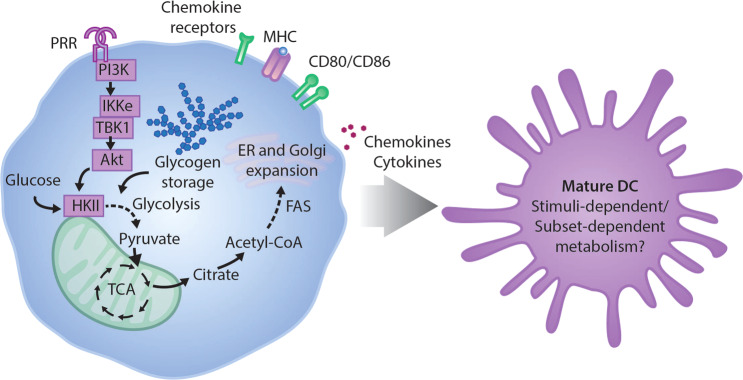
Metabolic reprogramming at early DC activation. Pattern recognition receptor (PRR) stimulation of immature DCs results in acute metabolic reprogramming leading to phosphoinositide 3-kinase (PI3K)/IκB-kinase ε (IKKε)/TANK-binding kinase 1 (TBK1)-dependent enhanced glycolysis fueled by intracellular glycogen storage. Glycolysis supports fatty acid synthesis (FAS), which promotes expansion of the endoplasmic reticulum (ER) and Golgi apparatus that are required for protein synthesis of costimulatory molecules, chemokines and cytokines. Following early activation, mature DCs obtain a cellular metabolism that may depend on the initial stimuli as well as the DC subset

Activation of GM-DCs or Flt3L-differentiated BMDCs with different TLR agonists has been shown to increase aerobic glycolysis [19]. This glycolytic burst has also been reported in response to other PRRs, including CLRs that recognize fungal-associated molecules such as *β*-glucan or zymosan [20, 21]. Thus, an immediate shift to glycolytic metabolism is believed to be a conserved response to PRR signaling in DCs (Fig. 2). Sustained glycolysis supports the survival of DCs under decreased OXPHOS, which was found to be suppressed due to robust nitric oxide (NO) production by inducible NO synthase (iNOS) [19, 22, 23]. Remarkably, the acute glycolytic increase is unaffected by NO production and fades to levels of unstimulated DCs after ~9 h [24]. Hence, activation of immature DCs can be subdivided into an early and a late phase with distinct metabolic regulation and requirements. NO-dependent late glycolysis was predominantly seen in GM-DCs and inflammatory moDCs and not in splenic cDCs, corresponding to iNOS expression selectively in inflammatory moDCs [24]. Despite the fact that NO derived from bystander cells such as macrophages can similarly inhibit OXPHOS in DCs [25], the iNOS-dependent glycolytic increase in vivo predominantly affects inflammatory moDCs [24]. Unlike the early glycolytic response, which is required for upregulation of costimulatory molecules, cytokine production and the T cell stimulatory capacity [19–23], late glycolysis is not required for DC maturation, as iNOS-inhibited DCs do not maintain glycolysis but retain their ability to upregulate costimulatory molecules [24]. It is thus likely that after the early glycolytic increase, individual DC subsets adapt distinct metabolic profiles depending on stimuli (Fig. 2).

#### Lipids in DC activation

Upon acute glycolytic activation, glucose can become a carbon source to support fatty acid synthesis (FAS). De novo FAS facilitates expansion of membranes of the endoplasmic reticulum (ER) and Golgi, which is required for the extensive increased biosynthesis of proinflammatory molecules during DC activation since inhibition of FAS diminishes DC maturation in response to lipopolysaccharides (LPS) [19] (Fig. 2). Furthermore, acetyl-CoA, the precursor for FAS, is a substrate for protein acetylation and a precursor for cholesterol synthesis. Cholesterol is an important component of cell membranes but can also become oxidized into oxysterols, which are regulators of lipid metabolism and can regulate transcription through nuclear hormone receptors such as liver X receptor. Cholesterol 25-hydroxylase (Ch25h) generates oxysterol 25-hydroxycholesterol (25-HC), and transcription of Ch25h is rapidly induced by TLR ligands poly(I:C) and LPS in CD11c^+^ GM-DCs in an IFN-I-dependent manner [26]. IFN-I-induced Ch25h was later shown to facilitate the suppression of enveloped viruses due to the direct antiviral properties of 25-HC [27]. Interestingly, the IFN-I response potentiates the activation of DCs and T cell priming and promotes pDC function via the enhancement of FAO [28, 29]. In addition to intrinsic lipid metabolism, DCs are often exposed to extracellular lipids in the tissue. Saturated fatty acids can activate APCs and upregulate costimulatory molecules and cytokine production, which is inhibited by polyunsaturated lipids [30]. Tumor-derived ceramide induces apoptosis in GM-DCs [31] and decreases the ability of human moDCs to take up soluble antigen [32], which agrees with the finding that lipid-overloaded DCs have a reduced ability to activate T cells [33].

#### Mitochondria in DC activation

**Fig. 3:**
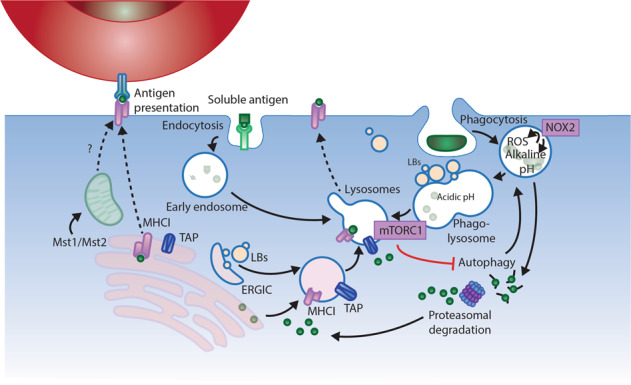
Metabolic regulation of antigen cross-presentation. Antigen cross-presentation requires uptake of extracellular antigen by either receptor-mediated endocytosis or phagocytosis. NADPH oxidase 2 (NOX2) is the major cellular ROS source, and it also maintains an alkaline pH in the early phagosome, which reduces antigen degradation, retaining antigen-peptide for MHC-I loading. Antigens are subsequently transferred to the cytosol and processed by proteasomal degradation before being loaded to MHC-I and presented on the cell surface. As an inhibitor of autophagy, mTORC1 reduces endogenous antigen uptake. Cross-presentation is supported by mitochondria driven by kinases Mst1/Mst2

To facilitate acute glycolytic activation, LPS induces the translocation of hexokinase II (HKII) to the mitochondrial membrane [19]. Moreover, the mitochondrial chaperone protein p32 interacts directly with the pyruvate dehydrogenase complex and facilitates glucose-derived citrate production required for TLR activation in GM-DCs [34]. Moreover, metabolic reprogramming is DC subset-specific and stimulation context-dependent. In support of this, it has been shown that IFN-I production by human PBMC-derived pDCs after activation of TLR9 is glycolysis-dependent, whereas IFN-I production after activation of RLR relies on OXPHOS. However, this is unique to pDCs, since human moDCs depend on glycolysis upon RLR activation [35]. Human pDCs also increase the expression of the mitochondrial biogenesis regulator peroxisome proliferator-activated receptor gamma coactivator 1-alpha (PGC1-α) and the mitochondrial fusion promoter Mfn2, supporting an increase in mitochondrial metabolism in response to TLR7/8 agonist pRNA. Conversely, human PBMC-derived myeloid CD1c^+^ DCs increase Drp1 expression to promote mitochondrial fission and reduce OXPHOS [36]. Intriguingly, a recent study identified that Mst1 and Mst2 kinases, which regulate mitochondrial trafficking next to phagosomes in macrophages, resulting in pathogen killing [37], are required for IL-12 production and antigen cross-presentation by cDC1s [12] (Fig. 3). In addition, pDCs can acquire the ability to cross-present antigens after TLR stimulation by increasing phagosomal pH to alkaline levels, resembling cDC1 cells [38]. This process required mitochondrial ROS, since reducing ROS with antioxidants or overexpressing a mitochondrial-targeted catalase diminished cross-presentation by pDCs [38]. Hence, mitochondrial integrity is vital for DC functions, particularly for antigen processing and presentation, which will be elaborated upon below.

#### mTORC1 and AMPK

mTORC1 is an important upstream regulator of glycolysis and FAS that can be activated by growth factor receptors or PRR signaling through the PI3K/Akt signaling pathway [39, 40]. mTORC1 further promotes protein translation, which is crucial for the survival and function of LPS-activated DCs [41]. In line with the central role of mTORC1 as a regulator of cell metabolism and translation, which is critical for DC function, it is clear that mTORC1 plays a significant role in guiding DC behavior. However, delineating the role of mTORC1 in DCs has proven complex, as studies have found mTORC1 inhibition in DCs to be both pro- and anti-inflammatory [42, 43]. Despite the fact that mTORC1 is a known glycolytic regulator, the early glycolytic switch in TLR-stimulated DCs was shown to be independent of mTORC1 and instead relied on the PI3K-mediated TBK1-IKKε-Akt pathway [19]. Glycolytic metabolism 24 h after LPS, on the other hand, was inhibited by the mTORC1 inhibitor rapamycin by inhibiting hypoxia-inducible factor-1alpha (HIF-1α)-iNOS activation [44]. Knockdown of HIF-1α, a downstream target of mTORC1, impaired the late glycolytic switch and maturation of LPS-stimulated GM-DCs, including IL-12 and TNF-α production [22]. Corresponding with a proinflammatory role for mTORC1 in DCs, rapamycin has been shown to inhibit maturation and promote a tolerogenic phenotype of human moDCs [45, 46], and a recent study showed that rapamycin impaired the production of proinflammatory cytokines in human moDCs in response to activation of RLR, a PRR that recognizes viral RNA [47]. In contrast, mTORC1 inhibition in CD11c^+^ GM-DCs and human PBMC-derived CD1c^+^ myeloid DCs potentiates the expression of the proinflammatory cytokines IL-12 and TNF-α in response to LPS. Moreover, constitutively active mTORC1 increases LPS-induced IL-10 but suppresses IL-12 [39, 40]. In agreement with this finding, rapamycin potentiates GM-DC-controlled activation of naïve CD8^+^ T cells [48]. Furthermore, knockout of the mTORC1 inhibitor tuberculosis sclerosis (TSC)1 in CD11c^+^ DCs results in decreased T cell proliferation [49] and a reduced number of activated CD8^+^ T cells [50], suggesting an impaired ability to prime naïve T cells in DCs with constitutively active mTORC1. Inhibition of mTORC1 has even been shown to prevent the effects of glucocorticoids in human PBMC-derived CD1c^+^ DCs [51], emphasizing the anti-inflammatory potential of mTORC1 in myeloid DCs. The discrepancies in mTORC1-related findings can partially be attributed to the different models used in DC studies, since Haidinger et al. showed that rapamycin had inhibitory effects on human moDCs differentiated with GM-CSF and IL-4 but stimulatory effects on human myeloid DCs isolated directly from blood [16].

AMPK is activated in energy-depleted situations by sensing AMP. AMPK activation controls catabolism and mitochondrial activity and can inhibit mTORC1 directly or through TSC2 activation [52]. AMPK knockdown with shRNAs followed by TLR stimulation potentiates IL-12p40 and CD86 expression in GM-DCs, whereas the AMPK activating compound 5-aminoimidazole-4-carboxamide ribonucleotide impairs both the glycolytic switching process and DC maturation after LPS treatment [23]. This indicates that AMPK is a negative regulator of DC activation. DC-specific knockout of the AMPK-activating kinase LKB1 also increases susceptibility to cancer and protects mice against asthma [13, 53], arguing for an anti-inflammatory role of AMPK. On the other hand, DC-specific knockout of LKB1 leads to splenic DCs with increased glycolytic metabolism and generates GM-DCs with enhanced capacity to prime T cells. Interestingly, both studies revealed that LKB1 knockout in DCs promotes the development of regulatory T cells (Tregs) and Th17 T cells, which was driven by LKB1 deletion in cDC2s. AMPK and mTORC1 are often reciprocally regulated, and LKB1-deficient cDCs have increased phosphorylation of S6 kinase, a downstream target of mTORC1 [13]. Inhibition of mTORC1 impairs the ability of cDC2s to generate Tregs [13], implying that LKB1 deficiency may promote inflammation in an mTORC1-dependent manner. Together, these findings emphasize that AMPK and mTORC1 have central roles in balancing DC pro- and anti-inflammatory responses in a highly context-dependent manner, and the complete mechanisms remain to be determined.

### Metabolic regulation of tolerogenic DCs

DCs are not only critical for initiating immune responses, but they are immensely important for maintaining tolerance. DCs also bestow peripheral tolerance by continuously presenting self-antigens as immature DCs in the absence of pro-inflammatory factors, silencing autoreactive T cell responses and promoting Tregs [19, 54]. Although immature DCs are tolerogenic, they can become activated when exposed to inflammatory stimuli. However, DC tolerance is sometimes maintained despite being in an inflammatory environment, which can be beneficial in autoinflammatory diseases but also lead to suppressed DC functions in the tumor microenvironment or during infection, facilitating immune evasion. Several factors, including IL-10, TGF-β, retinoic acids (RAs), glucocorticoids, vitamin D, and dexamethasone, can promote DC tolerance. Some of these factors are used to generate tolerogenic DCs in vitro.

In vivo, CD11c^+^ DC-specific β-catenin deletion substantially increases the inflammatory responses in intestinal DCs and worsens the symptoms of inflammatory bowel disease (IBD) [55]. Furthermore, tolerogenic DCs acquire the ability to produce RA, which promotes the formation of Tregs in a β-catenin-dependent manner [56]. Interestingly, tolerogenic cDC1s and cDC2s in visceral adipose tissue differ in their engagement of the canonical Wnt/β-catenin pathway [57, 58]. However, it remains elusive whether these differences are caused by subset-specific metabolic phenotypes or adaptation to the metabolic state in adipose tissues. Wnt5a activates the noncanonical Wnt pathway and has been shown to reduce IL-12 production by human moDCs [59, 60]. Moreover, Wnt5a increases FAO in CD11c^+^ GM-DCs and splenic cDCs by stimulating PPARγ pathways [60]. The active form of vitamin D 1,25-vitamine D3 (VitD3) also promotes the formation of tolerogenic DCs in vitro and in vivo [61], which may result from VitD3-induced enhancement of both glycolysis and OXPHOS and the activation of the PI3K/Akt/mTOR pathway [62]. In addition, repetitive LPS stimulation triggers the expression of indoleamine 2,3-dioxygenase (IDO), a metabolic enzyme responsible for metabolizing tryptophan into the immunosuppressive metabolite kynurenines, in splenic CD11c^+^ cDCs [63]. Interestingly, intratumoral treatment with IL-12 and GM-CSF also induces slow but stable IDO expression in DCs residing in the tumor-draining lymph node, correlating with a tolerogenic phenotype [64]. Moreover, both IFN-γ and TGF-β induce IDO expression in pDCs to orchestrate an immunosuppressive phenotype [65]. IDO1 expressed by splenic CD11c^+^ cDCs is activated in response to arginase-produced polyamines [66]. Therefore, it is believed that arginase and IDO1, two immunomodulatory metabolic enzymes, can cooperate to maintain long-term immunosuppressive effects in DCs.

### Metabolic regulation of antigen cross-presentation in DCs

DC maturation facilitates antigen proteolysis and efficient formation and trafficking of peptide-major histocompatibility complex (pMHC) to the plasma membrane [67]. Classically, endogenous antigens, such as proteins synthesized by virally infected cells, are presented on MHCI molecules for detection by CD8^+^ T cells. MHCII molecules generally bind peptides generated by lysosomal proteolysis in the endocytic and phagocytic pathways, and pMHCII is recognized by CD4^+^ T cells [68]. The activation of mTORC1 blocks autophagy, resulting in decreased endogenous antigen presentation of DCs [42]. In addition, IFN-γ also upregulates antigen processing and presentation as a result of suppressed mTORC1 activation and enhanced autophagy [69]. Furthermore, Pan et al. demonstrated that mTORC1 reduces MHCII mRNA transcription during DC maturation [70]. In contrast, mTOR activation positively supports lysosome tubulation, a process that facilitates peptide-loaded MHCII molecule trafficking from endolysosomes to the cell surface [71, 72]. Thus, these modulating effects controlled by mTORC1 optimize MHCII-mediated antigen presentation in DCs, and mTORC1 inhibition reduces antigen uptake at early time points while promoting antigen presentation at later time points.

In addition to conventional antigen presentation, cross-presentation is a specialized mechanism through which the antigen can be presented on MHCI, and it is critical for the induction of adaptive immunity against tumor cells and infectious pathogens [73]. The two most effective cross-presenting DCs identified are lymphoid-tissue resident cDC1s (CD11c^+^CD8α^+^XCR1^+^ DCs) and migratory cDC1s (CD11c^+^CD103^+^XCR1^+^ DCs) [74, 75]. Recent evidence indicates that DCs support their cross-presentation function through the establishment of distinct and tightly regulated metabolic processes, including glycolysis, OXPHOS and fatty acid metabolism [76, 77]. Here, we summarize the metabolic processes that have been shown to modulate cross-presentation (Fig. 3).

#### Lipid body

Lipid bodies (LBs), also known as lipid droplets, are neutral lipid storage organelles composed of a central core of cholesteryl esters and triglycerides surrounded by a single layer of phospholipids, which are thought to originate from ER membranes [78]. Lipid accumulation and synthesis of fatty acids have been shown to dictate DC functions, especially cross-presentation. These LBs accumulate in the cytosol and on DC phagosomes in an interferon inducible ER resident guanosine triphosphatase (GTPase) (Igtp)-dependent manner. Bougneres et al. showed that Igtp binds the LB coat component adipose differentiation-related protein (ADFP) to mediate the formation of LBs. Reducing LB amounts by inhibiting acyl CoA:diacylglycerol acyltransferase blocks the cross-presentation function of GM-DCs [79]. Additionally, liver-derived cDCs that contain high concentrations of intracellular lipids are more potent activators of proinflammatory T cell, NK cell and NKT cell responses, whereas liver-derived cDCs are more potent in facilitating the establishment of regulatory T cell-mediated tolerance if they express low levels of intracellular lipids [80]. In contrast to lipid synthesis and accumulation, Everts et al. reported that treatment with TOFA, an inhibitor of acetyl-CoA carboxylase 1, to abolish FAS, enhances cytokine and chemokine production in GM-DCs. Furthermore, treatment with C75, a fatty acid synthase inhibitor, results in an increased capacity of splenic cDCs to capture antigens and upregulates DC-dependent activation of CD4^+^ and CD8^+^ T cells in vivo [19]. However, some studies argue that FAS is dispensable for the activation and function of DCs [81]. In summary, the role of FAS in DC function, especially in regulating DC cross-presentation, remains elusive, and the reported discrepancy can result from the use of experimentally different stimuli that affect DC activation.

#### Optimal alkaline pH and ER metabolic homeostasis

The excellent ability of DCs to cross-present is largely attributed to their antigen processing capacity. As antigens are internalized by endocytosis or phagocytosis, they undergo gradual proteolytic degradation along their journey from early endosomes and phagosomes to lysosomes. Once in lysosomes, antigens are degraded by lysosomal proteases, which could destroy potential peptide epitopes crucial for T cell activation [68]. DCs circumvent this problem by expressing low levels of lysosomal proteases. Additionally, since most of these proteases function optimally at acidic pH, maintenance of a strongly alkaline pH in the cross-presentation compartment inhibits protease activity, thus preventing overt and limiting antigen degradation. To this end, alkalinization of phagosomes in DCs was attributed to recruitment, assembly and function of nicotinamide adenine dinucleotide phosphate (NADPH) oxidase-2 (NOX2) [82]. NOX2 is recruited to DC early phagosomes and mediates the sustained production of low levels of ROS, causing active and maintained alkalinization of the phagosomal lumen. NOX2-defective DCs showed enhanced phagosomal acidification and increased antigen degradation, causing impaired cross-presentation [82]. Therefore, NOX2 plays a critical role in conferring DCs the ability to function as specialized phagocytes adapted to process antigens rather than kill pathogens. NOX2 activity generates superoxide anions in the phagocytic lumen, which dismutate to produce hydrogen peroxide and other ROS. Furthermore, Ding et al. found that the lectin family member siglec-G recruited the phosphatase SHP-1, which dephosphorylated the NADPH oxidase component p47^phox^ and inhibited the activation of NOX2 on phagosomes. This resulted in excessive hydrolysis of exogenous antigens, which diminished the formation of MHCI-peptide complexes for cross-presentation [83]. Finally, NOX2 activity was shown to be crucial for cross-presentation, as genetic deletion of the NOX2 subunit gp91^phox^ abrogated cross-presentation. NOX2 reaches the cross-presenting compartment through the GTPases Rab27a and Rac2. In addition, VAMP-8 has also been reported to play a role in NOX2 recruitment and in mediating the cross-presentation of phagocytic antigens. To load exogenous peptides onto MHCI molecules, antigens are transported to the cytosol for proteasomal degradation. Antigenic peptides arising from proteasomal degradation are translocated into the ER lumen for antigen processing. Once in the ER, the peptides undergo amino-terminal trimming by ER-associated amino peptidases and are loaded on neosynthesized MHCI molecules by a complex composed of calreticulin, tapasin, and ERp57 [68]. DCs control endocytic functions that allow efficient antigen presentation, including the regulated transport of MHC molecules, the transport of antigen from endosomes and phagosomes into the cytosol, and the recruitment of ER proteins to phagosomes. All of these factors have been suspected to be controlled by metabolic processes involved in ER homeostasis in DCs.

#### DCs in the tumor microenvironment

**Fig. 4:**
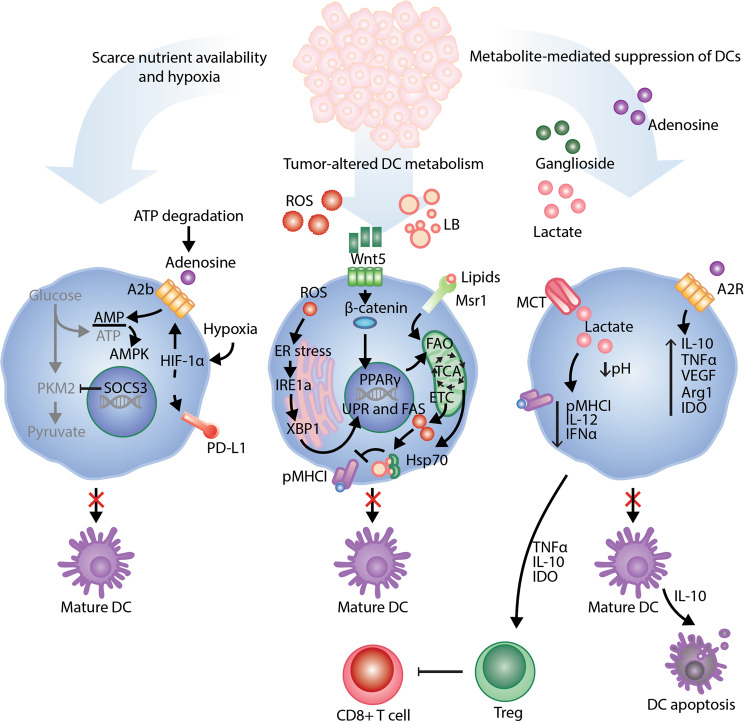
Metabolic regulation of DC function within the TME. DC metabolism is tightly linked with immune function and may be dysregulated within the TME in several ways. Competition between DCs and tumor cells for limiting resources can result in a lack of nutrient support for metabolic pathways essential to DC activation and maturation. Immunosuppressive cytokines, enzymes, and lipid bodies derived from tumors and tumor-associated cells can all alter metabolic pathways that support antitumor DC function while also driving alternative pathways associated with immune tolerance, including driving the differentiation of tumor-supporting Tregs. Immunosuppressive metabolites (adenosine, lactate, gangliosides and IDO generated by tumor cells) may trigger a shift in DCs from antitumor to protumor functionality. Abbreviations in this figure not defined in the main body of the text: PKM2, pyruvate kinase M2; A2R/A2b, A2 adenosine receptor. MCT, monocarboxylate transporters

It is now well established that tumor cells contain many antigens that can be recognized by the host immune system. Indeed, recent studies have demonstrated the important role of DCs in the generation of effective responses to immune checkpoint blockade (ICB) immunotherapy [84]. DCs are now known to contribute to various stages of the antitumor T cell response, from induction of an antigen-specific response in secondary lymphoid organs to recruitment of effector T cells into the tumor tissue and maintenance/restimulation of cytotoxic T lymphocytes within the tumor microenvironment (TME). Studies have indicated that tumor accumulation of cDC1s defined as Batf3-dependent DCs bearing the CD103 marker is associated with good prognosis and immune-mediated control across mouse and human species [85, 86]. The consequences of the decreased number of functionally competent DCs in patients with cancer are self-evident: a decreased number of APCs renders immune stimulation less effective. However, it is possible that other manifestations driving defective DC differentiation and activation affect antitumor immune responses, and DC metabolism may also be compromised within the TME in different settings (Fig. 4).

To support the biosynthetic and energy demands of rapid cell division, tumor cells characteristically undergo rewiring to a primarily glycolytic mode of metabolism not only in hypoxic environments where OXPHOS is naturally limited but also under normoxic conditions, a phenomenon known as Warburg metabolism [87]. Based on the aforementioned studies performed on BMDCs and monocyte-derived DCs in vitro, there are a number of potential and documented effects of glycolytically active tumor cells on DC function within the TME. Intriguingly, glucose deprivation and differential partitioning of nutrients in the TME have been suggested to modulate metabolic activities in tumor-infiltrating immune cells [88–90]. As a long-term commitment to glycolysis is needed to support ATP production in activated DCs, high AMP to ATP ratios resulting from insufficient metabolic fitness may in turn trigger AMPK-mediated downregulation of HIF-1α, a master transcriptional regulator of several enzymes essential to glycolytic metabolism, while antagonizing mTOR signaling. As described before, AMPK signaling activation possibly antagonizes anabolic metabolism, which impairs the DC effector function after stimulation with LPS [23, 91]. The PI3K/AKT/mTOR/HIF-1α signaling axis regulates the expression of enzymes necessary for glycogen synthesis. However, the functional consequences of commitment to aerobic glycolysis by DCs in the TME remain to be investigated because mTOR plays multifaceted roles in regulating DC function [43]. In murine models of melanoma as well as lung and ovarian cancers, tumor-associated DCs exhibited an increased expression of the suppressor of cytokine signaling-3 (SOCS3), which suppressed the activity of the pyruvate kinase M2 (PKM2) enzyme responsible for catalyzing the final step of glycolysis. In this study, SOCS3 was also found to limit the PKM2-driven antitumor efficacy of a DC-based vaccine against established Lewis lung carcinomas [92]. In addition to metabolic insufficiency induced by nutrient deprivation and partitioning, hypoxia is a common characteristic of the TME. Cancer cells can adapt to the diminished oxygen microenvironment, while immune cells are less capable of adapting to this microenvironment [76]. In human tumor moDCs, hypoxia-induced expression of HIF-1α activates AMP signaling by increasing A2b adenosine receptor mRNA [93]. Furthermore, HIF-1α dramatically increased the expression of programmed death-ligand 1 (PD-L1) in Gr1^+^CD11b^+^ myeloid cells by binding directly to the hypoxia-response element (HRE) in the PD-L1 proximal promoter [94]. This finding raised the interesting possibility that this process might also take place in tumor-infiltrating DCs (TIDCs) (Fig. 4).

In addition to limiting the availability of nutrients and other key resources for use by immune cell populations infiltrating the TME, the extreme metabolic demands of rapidly growing tumor cells also result in the accumulation of toxic byproducts that are detrimental to immune functions [95]. In this regard, the accumulation of lactic acids is a common factor impairing DC function. Lactate-derived DC dysfunction was first described in moDCs differentiated in melanoma cell line-derived multicellular tumor spheroids in a lactate dehydrogenase A inhibitor-sensitive manner [96]. GPR81, a G protein-coupled receptor (GPR) for lactic acids, can be found on both immune cells and cancer cells. The activation of GPR81 in cancer cells through autocrine lactic acid signaling promotes proliferation, drug resistance, and enhanced expression of PD-L1 [97]. In this context, lactic acids alter antigen presentation and the functional activity of DCs in a paracrine manner [97]. In addition to lactic acids, tumor cells can evade immune responses by producing high levels of RA, which directs intratumoral monocytes to differentiate into immunosuppressive macrophages rather than immunostimulatory dendritic cells via suppression of the DC-promoting transcription factor Irf4 [98].

Herber et al. reported that increased lipid deposition in TIDCs can be an important driver of tumor immune evasion [33]. The increase in lipid contents in TIDCs results from the class A macrophage scavenger receptor type 1 (Msr1, CD204)-mediated uptake of extracellular fatty acids, which further impairs DC-dependent stimulation of T-cell responses [33]. In support of this, Cubillos-Ruiz et al. recently showed that TIDCs exhibit activation of the unfolded protein response (UPR), as indicated by the presence of high levels of spliced X-box-binding protein 1 and robust lipid peroxidation [33]. As a result of ER stress and lipid peroxidation, TIDCs exhibit weaker antigen presentation ability. Notably, small-molecule inhibition of Msr1 restores the stimulatory capacity of these DCs. Despite these findings, how these alterations in lipid levels lead to impaired DC functionality remains uncertain. Until now, a more recent report described the molecular mechanism linking oxidized lipid bodies with the binding of the heat shock protein-70 chaperone and the defective transport of complexes of MHCI with peptide (pMHCI), which ultimately suppresses cross-presentation of antigens by DCs [99, 100]. These findings further suggest that perturbations in lipid metabolism and ER homeostasis can affect the antigen presentation ability of DCs and contribute to immune evasion.

Gangliosides are sialic acid-containing glycosphingolipids that have been implicated in the regulation of cellular proliferation and differentiation. An atypical expression pattern of gangliosides has been found in several tumor types, including neuroblastomas, medulloblastomas, melanomas, leukemias, lymphomas and breast tumors, compared with the corresponding normal tissues [101]. In addition to being expressed at the surface of tumor cell membranes, gangliosides are shed into the tumor microenvironment and eventually circulate in the peripheral blood. By using mouse and human neuroblastoma cell lines, researchers have shown that tumor-derived gangliosides inhibit the generation of DCs from mouse bone marrow cells or from human CD34^+^ precursors [102]. Monosialo-tetrahexosyl-ganglioside 3 (GM3) and disialoganglioside GD3 inhibit the phenotypic and functional differentiation of DCs and induce their apoptosis. In addition, these impaired DCs produces low amounts of IL-12 and large amounts of IL-10, which might hamper an efficient antitumor immune response [103]. Furthermore, ganglioside exposure alters APC function to induce Treg development or activation and may be related to ganglioside-dependent alterations in TLR signaling [104].

### Regulation of DCs by the gut microbiota

The intestinal immune system carries a formidable challenge in balancing immune responses toward pathogens penetrating the gut barrier as well as tolerance toward harmless food antigens and commensal microorganisms. DCs play a fundamental role in controlling this homeostasis. Dysregulated intestinal homeostasis can lead to chronic infections or inflammatory disorders, such as IBD. In addition, the gut microbiota is being increasingly appreciated as a regulator of the systemic immune systems, for example, by providing protection against melanoma cancers and airway inflammation [105–111]. Nonetheless, the means by which the gut microbiota controls host immunity remain incompletely understood. Here, we discuss the role of DCs in maintaining gut homeostasis and how the microbiota can regulate DC function, focusing on metabolic modulations.

#### Gut-associated DCs during steady-state and inflammation

**Fig. 5:**
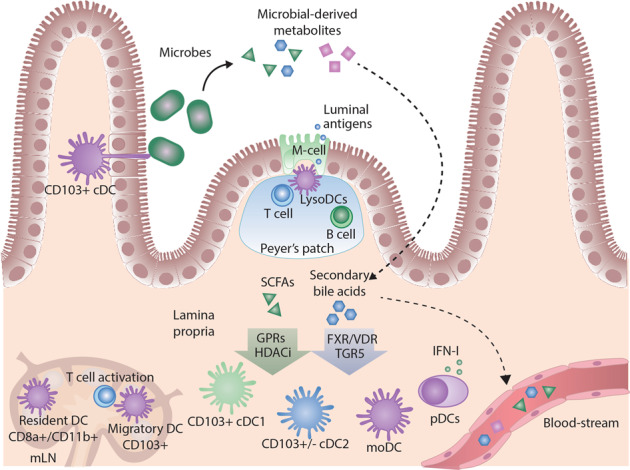
Modulation of DC function by gut microbial metabolites. Intestinal DCs sample luminal antigen in the form of commensal bacteria or ingested food from the gut by either penetrating epithelial cell-cell junctions and collecting antigen directly or by receiving antigen by M-cells transporting antigen to DCs in the lymphoid tissue Peyer’s patch. Peyer’s patch-localized DCs present antigens to T cells directly, directing CD4+ T cell differentiation and supporting IgA production by B cells. CD103+ cDCs are the major migratory DCs that traffic antigens to the mesenteric lymph node (mLN) to present antigens to naïve T cells. The gut microbiota produces an abundance of microbially modified metabolites that can modulate immune cells directly in the gut or enter the bloodstream and have systemic effects. The most studied immune-modulating gut-derived metabolites are short-chain fatty acids (SCFAs) that can activate G-protein coupled receptors (GPRs) or act as histone deacetylase inhibitors (HDACis). Secondary bile acids can activate farnesoid X receptor (FXR), vitamin D receptor (VDR) and Takeda G protein coupled receptor 5 (TGR5), among others

Gut-associated DCs are indispensable for maintaining gut homeostasis. Deletion of pre-DCs in Zbtb46-DTR mice has been shown to disrupt oral tolerance toward orally supplemented ovalbumin antigen [112], and ablation of CD11c^+^ DCs leads to spontaneous autoimmunity and IBD [113]. However, excessive production of the proinflammatory cytokines IL-12p40 and IL-23 by DCs can promote intestinal inflammation [114]; hence, uncontrolled DC activation disrupts gut homeostasis. Intestinal DCs reside in gut-associated lymphoid organs comprising Peyer’s patches (PPs) and mesenteric lymph nodes (mLNs) but are also distributed in connective tissues beneath the gut epithelium called the lamina propria (LP). DCs can obtain gut luminal antigens in two ways. Specialized epithelial M cells that transport luminal antigens to PPs, where resident DCs high on lysosymes take up the antigens and prime naïve T cells [115, 116]. Alternatively, migratory CD103^+^ cDCs can be used to sample luminal antigens themselves by penetrating epithelial tight junctions during steady-state conditions and promote tolerance by activating Tregs and producing TGF-β and RA [117–119] (Fig. 5).

The LP contains DC subsets derived from circulating monocytes (moDCs) or directly from bone marrow pre-cDCs [120, 121]. moDCs are not sufficient for maintaining oral tolerance [112] or tolerance in a DSS-induced colitis model lacking CD103^+^ cDCs [120]. Instead, moDCs mainly induce Th17 responses [122]. CD103^+^ cDCs display a more tolerogenic gene expression pattern [112], indicating that gut-associated DC subsets have distinct roles in balancing tolerance and inflammation. Nevertheless, CD103^+^ DCs also participate in activating immune responses, as they have been shown to be the first to transport Salmonella from the intestinal tract to the mLN by increasing CCR7 expression [121]. How DCs selectively induce activating immune responses against pathogenic bacteria is still not fully understood. Human CD103^+^ cDC2s were shown to promote a proinflammatory Th17 response when recognizing IgA immune complexes, which are only present in LP after pathological infections [123], suggesting that environmental factors such as IgA immune complexes could be an indicator of harmful infection and thus mount an inflammatory response. Hansen et al. additionally showed that IgA immune complex-induced cytokine production required Syk-PI3K-TBK1-IKKε glycolytic reprogramming [123], indicating that intestinal DCs require a glycolytic switch when transitioning from tolerogenic to inflammatory and that modulation of DC metabolism may alter intestinal DC functionality.

#### Gut-microbiota-controlled systemic DC immunity

The microbiota influences the maturation of neonatal DC compartments and has been shown to promote the immunosuppressive functions of splenic pre-cDC1s within the first week of life [124]. Microbiota exposure of neonates from antibiotic-treated mothers kick-starts pre-cDC1 differentiation into cDC1s, at least partially facilitated by microbiota-induced TNF production from myeloid cells [125]. In addition, splenic DCs from germ-free mice fail to produce IFN-I due to suppressive histone modifications at the cytokine promotors [126]. The gut microbiota is not only instructive in the formation of the neonatal immune system, but its immune regulatory properties can be harnessed by fecal microbiota transplants (FMTs) in adults, aiding in the treatment of antibiotic-resistant infections, type 2 diabetes, and cancer, among others [127–130]. In a very elegant study, Geva-Zatorsky et al. examined single human microbiota-derived species in monocultures in mice, discovering the extensive modulating effect the microbiota has on gut-resident immune cells. In particular, colonic pDCs were heavily regulated by different colonization, and pDC frequencies correlated with enriched transcripts of lipid and protein metabolism pathways in whole-tissue transcriptomics [131]. pDCs are the major source of IFN-I and furthermore possess unique trafficking properties compared with cDCs. pDCs circulate the blood during steady-state conditions, indicating a possible way of maintaining systemic IFN-I levels. However, to egress from BM, pDCs require CCL2, which is constitutively expressed in a microbiota-dependent manner [132]. Moreover, the microbiota activates TLR4-TRIF signaling in LP-localized DCs, which promotes IFN-β production and systemic interferon stimulated gene (ISG) responses [133]. It was recently shown that microbiota-induced steady-state IFN-I signaling from pDCs allows cDCs to prime T cells in a metabolically dependent manner [134]. Schaupp et al. showed that Ifnar-deficient cDCs had altered histone modifications and reduced mitochondrial metabolism, similar to cDCs from germ-free mice [134]. This indicates that the commensal microbiota supports systemic IFN-I signaling, imprinting cDCs metabolically and epigenetically, which likely facilitates the enhanced T cell priming seen in mice with full microbiota.

Two clinical trials with FMT from patients responding to ICB anti-PD-1 therapy were recently shown to enhance the response to therapy in previously nonresponding melanoma patients [127, 129]. This achievement is based on a body of work demonstrating that the response to ICB therapy is dependent on the composition of the gut microbiota and that transfer of fecal microbiota or specific commensal strains could enhance the response to ICB therapy in mice [105–108, 135]. Interestingly, DC tumor infiltration has been shown to promote the response to ICB therapy [75, 136], suggesting that the microbiota could enhance the response to ICB therapy by promoting DC tumor infiltration, cross-presentation and cytokine production. Accordingly, myeloid DCs were enriched in PBMCs of ICB responding patients [107], and Sivan et al. reported augmented DC tumor infiltration and enhanced CD8^+^ T cell priming in mice with a full microbiota compared with antibiotics-treated or germ-free mice [105]. Moreover, depletion of cDCs after tumor engraftment of colonized Zbtb46-DTR bone marrow chimeric mice revealed that cDCs are required for the effect of ICB-promoting bacteria [137]. Finally, a defined 11 strain consortium of commensals that promote antitumor immunity also increased MHCI expression on LP-associated DCs as well as systemically increased IFN-γ^+^ CD8^+^ T cells, indicating enhanced CD8^+^ T cell priming. This effect required both cDC1s and cDC2s, since *Batf3-, Xcr1-, Irf4-*, or *Notch*-deficient mice all abolished 11-strain-induced IFN-γ^+^ CD8^+^ T cells [138]. Thus, the precise role of DCs in linking the microbiota to enhanced antitumor immunity remains to be uncovered.

The TME comprises bacteria as well, and characterizations of the tumor microbiome have emerged in recent years, revealing intracellular bacteria in both tumor and immune cells [139, 140]. The presence of bacteria within the tumor suggests that these can influence tumor immunity directly [141, 142]. Indeed, it has been shown that gut microbial bacteria can accumulate in subcutaneous tumors and regulate the response to anti-CD47 therapy by controlling the IFN-I response by CD11c^+^ DCs [143]. Hence, the gut microbiota can control tumors at distal sites by regulating circulating immune cells, modulating the tumor microbiome, or producing compounds that enter the circulation.

#### Gut microbiota-derived metabolites

The gut microbiota comprises trillions of microorganisms that possess unique genes, some of which encode proteins that metabolize food intake undigestible by the host and others that modify host-derived metabolites in a distinct manner. Certain microbiota-derived metabolites have immune modulating functions that directly affect intestinal immune cells, and some enter the circulation, presenting a potential way for the microbiota to modulate systemic immunity. DC infiltration and antigen uptake in the lung are, for example, reduced by microbiota-sulfated p-cresol, resulting in reduced airway inflammation [111]. Conversely, the fecal levels of the gut-derived lipid 12,13-diHOME have been shown to be associated with the risk of developing allergic inflammation and were recently found to increase pulmonary inflammation and reduce Treg development in the lungs [144], emphasizing that microbiota-derived metabolites can have both pro- and anti-inflammatory effects. Human DCs responded to 12,13-diHOME by altering PPARγ-regulated genes and reducing anti-inflammatory cytokine production and Treg activation in vitro [144], suggesting that 12,13-diHOME-induced inflammation could be facilitated by breaking DC-controlled tolerance. Short-chain fatty acids (SCFAs) are also highly abundant in the gut and are produced from gut microbial fermentation of fibers undigestible by the human digestive system. SCFAs acetate, propionate, and butyrate, with two, three, and four carbon atoms, respectively, are known to have immune modulating properties. Butyrate has anti-inflammatory effects and promotes Tregs in the gut, maintaining gut homeostasis [145]. Several studies have uncovered the SCFA-mediated regulation of T cells, but less is known about the mechanisms behind SCFA-mediated regulation of DCs. Most evidence points to an anti-inflammatory effect of SCFAs on DCs. Dietary fibers and SCFAs protect against airway inflammation by poorly increasing antigen-presenting DCs in airways, dampening the Th2 response [109]. Butyrate and propionate have been shown to prevent LPS-induced maturation markers and the production of proinflammatory chemokines and cytokines by human moDCs [146, 147]. High levels of butyrate and propionate are further associated with resistance to CTLA4 blockade in metastatic melanoma patients, and butyrate supplementation reduced anti-CTLA4-induced DC maturation and CD8^+^ T cell priming in a syngeneic tumor mouse model [148]. In line with this, depletion of gram-positive bacteria using vancomycin increased antigen presentation by CD103^+^ DCs and potentiated the antitumor response in a melanoma model, which was reversed by supplementation with butyrate [149]. In contrast, previous studies have reported that dietary fiber inulin, which is fermented to SCFAs, enhances DC number and proportion in PPs of rats [150, 151]. Inulin further enhanced cDC1 tumor infiltration and CD8^+^ T cell antitumor response [152], as well as provided protection against influenza infections [153], supporting a DC-activating effect of SCFAs. These inconsistencies may be explained by the different models used but could also be attributed to the multiple mechanisms of SCFAs. Mechanistically, SCFAs can regulate host cells in three ways. First, they can bind and activate GPRs. Second, they can enter host cell metabolism as metabolites. Third, butyrate and propionate have histone deacetylase inhibitor (HDACi) effects, modifying the epigenetic landscape of the host cells. Butyrate has been shown to induce an epigenetic program in macrophages, which reduces glycolysis and mTOR and promotes autophagosome-lysosome maturation [154], potentially promoting antigen presentation. However, it is not known whether butyrate has a similar effect on DCs. Interestingly, whether butyrate acts on the host cell as a metabolite or as an HDACi depends on host cell metabolism in cancerous colonocytes, resulting in highly distinctive outcomes and epigenetic changes [155]. If the same applies to DCs, butyrate can have distinct effects on individual DC subsets depending on their maturation stage.

The gut microbiota also communicates with the liver through modification of liver-synthesized bile acids. Primary bile acids produced by the host in the liver are modified by the gut microbiota to become secondary bile acids. A fraction of the secondary bile acids re-enters the circulation and can thus regulate host cells systemically. Microbiota-derived secondary bile acids were recently discovered to have substantial influence on the intestinal Treg/Th17 balance [156–158]. The most common receptors with the highest affinity for bile acids are the surface receptor Takeda G-protein receptor 5, the nuclear receptor Farnesoid-x-receptor (FXR) and the vitamin D receptor (VDR) [159]. DCs express high levels of VDR, and VDR agonists have previously been shown to target myeloid DCs in particular [160], yet VDR-deficient DCs in VDR-flox CD11c-cre mice had no effect on bile acid-induced Tregs [158]. FXR agonists in DSS-induced colitis increase splenic DCs, promote Tregs, and protect mice from IBD [161, 162]. Intriguingly, ablation of FXR in DCs enhanced Tregs in a manner comparable to the secondary bile acid isoDCA [156], suggesting that isoDCA may enhance Tregs by modulating DCs, perhaps in an FXR antagonistic manner. The secondary bile acid DCA was recently shown to restore tonic pDC production of IFN-I, maintain the ISG signature in circulating monocytes of germ-free or antibiotic-treated mice, and rescue the response to Chikungunya virus [163]. Hence, bile acids and their microbiota-modified derivatives control gut homeostasis as well as systemic immunity. The gut microbiota influences immune cell epigenetics, for example, through the production of SCFAs, as mentioned above. Moreover, the gut microbiome can regulate the epigenome of splenic cDCs through regulation of tonic IFN-I production, instructing cDCs for future challenges [134]. Cell metabolism and epigenetics are tightly regulated; hence, future studies on how the gut microbiota influences DC metabolism and epigenetics may lead to an advanced understanding of DC biology.

## Conclusion

The field of immunometabolism is just emerging and has become one of the most exciting research areas. Our review focuses on studies uncovering new molecular processes and metabolic pathways that control the development, activation and function of dendritic cells. With DCs playing a central role in launching appropriate immune responses, they also pose a promising target for therapeutic interventions. Expansion of DC development in combination with TLR ligands or CD40 agonists has been shown to be applicable in cancer therapy in mouse models [75, 164]. However, expanding the DC population also poses the risk of increasing the reservoir for intracellular infections, as seen by the impaired response to *Listeria monocytogenes* and *Mycobacterium tuberculosis* [165]. DC vaccines present the most promising approach in cancer therapy, where moDCs are activated and loaded with peptides before being transferred back to the patient. With the expanding knowledge on how to manipulate DC activation, DC vaccines can both be used to activate antitumor immune responses and suppress autoimmune diseases by transferring tolerogenic moDCs [166]. Several clinical trials involving tolerogenic DCs from either bone marrow or blood are currently ongoing and are nicely summarized by others [166]. A full comprehension of DC metabolism could present new ways of optimizing DC vaccines by enhancing their metabolic potential to fight cancer.

## References

[1] Murphy TL, Grajales-Reyes GE, Wu X, Tussiwand R, Briseño CG, Iwata A (2016). Transcriptional control of dendritic cell development. Annu Rev Immunol.

[2] Merad M, Sathe P, Helft J, Miller J, Mortha A (2013). The dendritic cell lineage: ontogeny and function of dendritic cells and their subsets in the steady state and the inflamed setting. Annu Rev Immunol.

[3] Liu K, Victora GD, Schwickert TA, Guermonprez P, Meredith MM, Yao K (2009). In vivo analysis of dendritic cell development and homeostasis. Science.

[4] Rodrigues PF, Alberti-Servera L, Eremin A, Grajales-Reyes GE, Ivanek R, Tussiwand R (2018). Distinct progenitor lineages contribute to the heterogeneity of plasmacytoid dendritic cells. Nat. Immunol.

[5] Dress RJ, Dutertre CA, Giladi A, Schlitzer A, Low I, Shadan NB (2019). Plasmacytoid dendritic cells develop from Ly6D+ lymphoid progenitors distinct from the myeloid lineage. Nat Immunol.

[6] Villani AC, Satija R, Reynolds G, Sarkizova S, Shekhar K, Fletcher J, et al. Single-cell RNA-seq reveals new types of human blood dendritic cells, monocytes, and progenitors. Science. 2017;356:eaah4573 10.1126/science.aah4573 PMC5775029 28428369

[7] Zilionis R, Engblom C, Pfirschke C, Savova V, Zemmour D, Saatcioglu HD (2019). Single-cell transcriptomics of human and mouse lung cancers reveals conserved myeloid populations across individuals and species. Immunity..

[8] Maier B, Leader AM, Chen ST, Tung N, Chang C, LeBerichel J (2020). A conserved dendritic-cell regulatory program limits antitumour immunity. Nature..

[9] McKenna HJ, Stocking KL, Miller RE, Brasel K, De Smedt T, Maraskovsky E (2000). Mice lacking flt3 ligand have deficient hematopoiesis affecting hematopoietic progenitor cells, dendritic cells, and natural killer cells. Blood..

[10] Sathaliyawala T, O’Gorman WE, Greter M, Bogunovic M, Konjufca V, Hou ZE (2010). Mammalian target of rapamycin controls dendritic cell development downstream of Flt3 ligand signaling. Immunity..

[11] Kratchmarov R, Viragova S, Kim MJ, Rothman NJ, Liu K, Reizis B (2018). Metabolic control of cell fate bifurcations in a hematopoietic progenitor population. Immunol. Cell Biol.

[12] Du X, Wen J, Wang Y, Karmaus PWFF, Khatamian A, Tan H (2018). Hippo/Mst signalling couples metabolic state and immune function of CD8α+ dendritic cells. Nature.

[13] Pelgrom LR, Patente TA, Sergushichev A, Esaulova E, Otto F, Ozir-Fazalalikhan A (2019). LKB1 expressed in dendritic cells governs the development and expansion of thymus-derived regulatory T cells. Cell Res.

[14] Pearce EJ, Everts B (2015). Dendritic cell metabolism. Nat. Rev. Immunol.

[15] Xu Y, Zhan Y, Lew AM, Naik SH, Kershaw MH (2007). Differential development of murine dendritic cells by GM-CSF versus Flt3 ligand has implications for inflammation and trafficking. J. Immunol.

[16] Haidinger M, Poglitsch M, Geyeregger R, Kasturi S, Zeyda M, Zlabinger GJ (2010). A versatile role of mammalian target of rapamycin in human dendritic cell function and differentiation. J Immunol..

[17] Del Prete A, Zaccagnino P, Di Paola M, Saltarella M, Oliveros Celis C, Nico B (2008). Role of mitochondria and reactive oxygen species in dendritic cell differentiation and functions. Free Radic Biol Med..

[18] Zaccagnino P, Saltarella M, Maiorano S, Gaballo A, Santoro G, Nico B (2012). An active mitochondrial biogenesis occurs during dendritic cell differentiation. Int J Biochem. Cell Biol.

[19] Everts B, Amiel E, Huang SC, Smith AM, Chang CH, Lam WY (2014). TLR-driven early glycolytic reprogramming via the kinases TBK1-IKKɛ supports the anabolic demands of dendritic cell activation. Nat Immunol.

[20] Thwe PM, Fritz DI, Snyder JP, Smith PR, Curtis KD, O’Donnell A (2019). Syk-dependent glycolytic reprogramming in dendritic cells regulates IL-1β production to β-glucan ligands in a TLR-independent manner. J Leukoc Biol.

[21] Guak H, Al Habyan S, Ma EH, Aldossary H, Al-Masri M, Won SY (2018). Glycolytic metabolism is essential for CCR7 oligomerization and dendritic cell migration. Nat Commun..

[22] Jantsch J, Chakravortty D, Turza N, Prechtel AT, Buchholz B, Gerlach RG (2008). Hypoxia and hypoxia-inducible factor-1α modulate lipopolysaccharide-induced dendritic cell activation and function. J Immunol.

[23] Krawczyk CM, Holowka T, Sun J, Blagih J, Amiel E, DeBerardinis RJ (2010). Toll-like receptor-induced changes in glycolytic metabolism regulate dendritic cell activation. Blood.

[24] Everts B, Amiel E, Van Der Windt GJW, Freitas TC, Chott R, Yarasheski KE (2012). Commitment to glycolysis sustains survival of NO-producing inflammatory dendritic cells. Blood.

[25] Amiel E, Everts B, Fritz D, Beauchamp S, Ge B, Pearce EL (2014). Mechanistic target of Rapamycin inhibition extends cellular lifespan in dendritic cells by preserving mitochondrial function. J Immunol.

[26] Park K, Scott AL (2010). Cholesterol 25-hydroxylase production by dendritic cells and macrophages is regulated by type I interferons. J Leukoc Biol.

[27] Liu SY, Aliyari R, Chikere K, Li G, Marsden MD, Smith JK (2013). Interferon-inducible cholesterol-25-hydroxylase broadly inhibits viral entry by production of 25-hydroxycholesterol. Immunity..

[28] Wu D, Sanin DE, Everts B, Chen Q, Qiu J, Buck MD (2016). Type 1 interferons induce changes in core metabolism that are critical for immune function. Immunity..

[29] Basit F, de Vries IJM (2019). Dendritic cells require PINK1-mediated phosphorylation of BCKDE1α to promote fatty acid oxidation for immune function. Front Immunol.

[30] Weatherill AR, Lee JY, Zhao L, Lemay DG, Youn HS, Hwang DH (2005). Saturated and polyunsaturated fatty acids reciprocally modulate dendritic cell functions mediated through TLR4. J Immunol.

[31] Kanto T, Kalinski P, Hunter OC, Lotze MT, Amoscato AA (2001). Ceramide mediates tumor-induced dendritic cell apoptosis. J Immunol..

[32] Sallusto F, Nicolò C, De Maria R, Corinti S, Testi R (1996). Ceramide inhibits antigen uptake and presentation by dendritic cells. J Exp Med..

[33] Herber DL, Cao W, Nefedova Y, Novitskiy SV, Nagaraj S, Tyurin VA (2010). Lipid accumulation and dendritic cell dysfunction in cancer. Nat Med.

[34] Gotoh K, Morisaki T, Setoyama D, Sasaki K, Yagi M, Igami K (2018). Mitochondrial p32/C1qbp is a critical regulator of dendritic cell metabolism and maturation. Cell Rep.

[35] Fekete T, Sütö MI, Bencze D, Mázló A, Szabo A, Biro T (2018). Human plasmacytoid and monocyte-derived dendritic cells display distinct metabolic profile upon RIG-I activation. Front Immunol.

[36] Basit F, Mathan T, Sancho D, De Vries JM (2018). Human dendritic cell subsets undergo distinct metabolic reprogramming for immune response. Front Immunol.

[37] Geng J, Sun X, Wang P, Zhang S, Wang X, Wu H (2015). Kinases Mst1 and Mst2 positively regulate phagocytic induction of reactive oxygen species and bactericidal activity. Nat Immunol.

[38] Oberkampf M, Guillerey C, Mouriès J, Rosenbaum P, Fayolle C, Bobard A (2018). Mitochondrial reactive oxygen species regulate the induction of CD8+ T cells by plasmacytoid dendritic cells. Nat Commun..

[39] Ohtani M, Nagai S, Kondo S, Mizuno S, Nakamura K, Tanabe M (2008). Mammalian target of rapamycin and glycogen synthase kinase 3 differentially regulate lipopolysaccharide-induced interleukin-12 production in dendritic cells. Blood..

[40] Weichhart T, Costantino G, Poglitsch M, Rosner M, Zeyda M, Stuhlmeier KM (2008). The TSC-mTOR signaling pathway regulates the innate inflammatory response. Immunity.

[41] Lelouard H, Schmidt EK, Camosseto V, Clavarino G, Ceppi M, Hsu HT (2007). Regulation of translation is required for dendritic cell function and survival during activation. J Cell Biol.

[42] Sukhbaatar N, Hengstschläger M, Weichhart T (2016). mTOR-Mediated regulation of dendritic cell differentiation and function. Trends Immunol.

[43] Snyder JP, Amiel E (2019). Regulation of dendritic cell immune function and metabolism by cellular nutrient sensor mammalian target of rapamycin (mTOR). Front Immunol.

[44] Lawless SJ, Kedia-Mehta N, Walls JF, McGarrigle R, Convery O, Sinclair LV (2017). Glucose represses dendritic cell-induced T cell responses. Nat Commun.

[45] Monti P, Mercalli A, Eugenio Leone B, Valerio DC, Allavena P, Piemonti L (2003). Rapamycin impairs antigen uptake of human dendritic cells. Transplantation..

[46] Hackstein H, Taner T, Zahorchak AF, Morelli AE, Logar AJ, Gessner A (2003). Rapamycin inhibits IL-4-induced dendritic cell maturation in vitro and dendritic cell mobilization and function in vivo. Blood..

[47] Fekete T, Ágics B, Bencze D, Bene K, Szántó A, Tarr T, et al. Regulation of RLR-mediated antiviral responses of human dendritic cells by mTOR. Front Immunol. 2020;11:572960. 10.3389/fimmu.2020.572960 PMC7516067 33013932

[48] Amiel E, Everts B, Freitas TC, King IL, Curtis JD, Pearce EL (2012). Inhibition of mechanistic target of rapamycin promotes dendritic cell activation and enhances therapeutic autologous vaccination in mice. J. Immunol.

[49] Wang Y, Huang G, Zeng H, Yang K, Lamb RF, Chi H Tuberous sclerosis 1 (Tsc1)-dependent metabolic checkpoint controls development of dendritic cells. Proc Natl Acad Sci USA. 2013;110:E4894–903. 10.1073/pnas.1308905110 PMC3864282 24282297

[50] Shi L, Chen X, Zang A, Li T, Hu Y, Ma S (2019). TSC1/mTOR-controlled metabolic-epigenetic cross talk underpins DC control of CD8+ T-cell homeostasis. PLoS Biol.

[51] Weichhart T, Haidinger M, Katholnig K, Kopecky C, Poglitsch M, Lassnig C (2011). Inhibition of mTOR blocks the anti-inflammatory effects of glucocorticoids in myeloid immune cells. Blood..

[52] Saravia J, Raynor JL, Chapman NM, Lim SA, Chi H (2020). Signaling networks in immunometabolism. Cell Res.

[53] Wang Y, Du X, Wei J, Long L, Tan H, Guy C (2019). LKB1 orchestrates dendritic cell metabolic quiescence and anti-tumor immunity. Cell Res.

[54] Iberg CA, Jones A, Hawiger D (2017). Dendritic cells as inducers of peripheral tolerance. Trends Immunol.

[55] Manicassamy S, Reizis B, Ravindran R, Nakaya H, Salazar-Gonzalez RM, Wang YC (2010). Activation of β-catenin in dendritic cells regulates immunity versus tolerance in the intestine. Science..

[56] Hong Y, Manoharan I, Suryawanshi A, Majumdar T, Angus-Hill ML, Koni PA (2015). β-catenin promotes regulatory T-cell responses in tumors by inducing vitamin a metabolism in dendritic cells. Cancer Res.

[57] Macdougall CE, Wood EG, Loschko J, Scagliotti V, Cassidy FC, Robinson ME (2018). Visceral adipose tissue immune homeostasis is regulated by the crosstalk between adipocytes and dendritic cell subsets. Cell Metab.

[58] LaMarche NM, Lynch L (2018). Adipose dendritic cells come out of hiding. Cell Metab.

[59] Valencia J, Martínez VG, Hidalgo L, Hernández-López C, Canseco NM, Vicente Á (2015). Wnt5a signaling increases IL-12 secretion by human dendritic cells and enhances IFN-γ production by CD4+ T cells. Immunol Lett.

[60] Zhao F, Xiao C, Evans KS, Theivanthiran T, DeVito N, Holtzhausen A (2018). Paracrine Wnt5a-β-catenin signaling triggers a metabolic program that drives dendritic cell tolerization. Immunity.

[61] Kleijwegt FS, Laban S, Duinkerken G, Joosten AM, Koeleman BPC, Nikolic T (2011). Transfer of regulatory properties from tolerogenic to proinflammatory dendritic cells via induced autoreactive regulatory T cells. J Immunol.

[62] Ferreira GB, Vanherwegen AS, Eelen G, Gutiérrez ACF, VanLommel L, Marchal K (2015). Vitamin D3 induces tolerance in human dendritic cells by activation of intracellular metabolic pathways. Cell Rep.

[63] Fallarino F, Pallotta MT, Matino D, Gargaro M, Orabona C, Vacca C (2015). LPS-conditioned dendritic cells confer endotoxin tolerance contingent on tryptophan catabolism. Immunobiology..

[64] Li Q, Harden JL, Anderson CD, Egilmez NK (2016). Tolerogenic phenotype of IFN-γ–Induced IDO + dendritic cells is maintained via an autocrine IDO–Kynurenine/AhR–IDO Loop. J Immunol.

[65] Pallotta MT, Orabona C, Volpi C, Vacca C, Belladonna ML, Bianchi R (2011). Indoleamine 2,3-dioxygenase is a signaling protein in long-term tolerance by dendritic cells. Nat Immunol.

[66] Mondanelli G, Bianchi R, Pallotta MT, Orabona C, Albini E, Iacono A (2017). A relay pathway between arginine and tryptophan metabolism confers immunosuppressive properties on dendritic cells. Immunity..

[67] Trombetta ES, Ebersold M, Garrett W, Pypaert M, Mellman I (2003). Activation of lysosomal function during dendritic cell maturation. Science..

[68] Blum JS, Wearsch PA, Cresswell P (2013). Pathways of antigen processing. Annu Rev Immunol..

[69] Su X, Yu Y, Zhong Y, Giannopoulou EG, Hu X, Liu H (2015). Interferon-γ regulates cellular metabolism and mRNA translation to potentiate macrophage activation. Nat. Immunol.

[70] Pan H, O’Brien TF, Wright G, Yang J, Shin J, Wright KL (2013). Critical role of the tumor suppressor tuberous sclerosis complex 1 in dendritic cell activation of CD4 T cells by promoting MHC class II expression via IRF4 and CIITA. J Immunol.

[71] Saric A, Hipolito VEB, Kay JG, Canton J, Antonescu CN, Botelho RJ (2016). mTOR controls lysosome tubulation and antigen presentation in macrophages and dendritic cells. Mol Biol Cell.

[72] Vyas JM, Kim Y-M, Artavanis-Tsakonas K, Love JC, Van der Veen AG, Ploegh HL (2007). Tubulation of class II MHC compartments is microtubule dependent and involves multiple endolysosomal membrane proteins in primary dendritic cells. J Immunol.

[73] Joffre OP, Segura E, Savina A, Amigorena S (2012). Cross-presentation by dendritic cells. Nat Rev Immunol.

[74] Veglia F, Gabrilovich DI (2017). Dendritic cells in cancer: the role revisited. Curr Opin Immunol.

[75] Salmon H, Idoyaga J, Rahman A, Leboeuf M, Remark R, Jordan S (2016). Expansion and activation of CD103+ dendritic cell progenitors at the tumor site enhances tumor responses to therapeutic PD-L1 and BRAF inhibition. Immunity.

[76] Giovanelli P, Sandoval TA, Cubillos-Ruiz JR (2019). Dendritic cell metabolism and function in tumors. Trends Immunol.

[77] Du X, Chapman NM, Chi H (2018). Emerging roles of cellular metabolism in regulating dendritic cell subsets and function. Front Cell Dev Biol.

[78] Fujimoto T, Ohsaki Y (2006). Proteasomal and autophagic pathways converge on lipid droplets. Autophagy.

[79] Bougnères L, Helft J, Tiwari S, Vargas P, Chang BH-J, Chan L (2009). A role for lipid bodies in the cross-presentation of phagocytosed antigens by MHC class I in dendritic cells. Immunity..

[80] Ibrahim J, Nguyen AH, Rehman A, Ochi A, Jamal M, Graffeo CS (2012). Dendritic cell populations with different concentrations of lipid regulate tolerance and immunity in mouse and human liver. Gastroenterology..

[81] Stüve P, Minarrieta L, Erdmann H, Arnold-Schrauf C, Swallow M, Guderian M (2018). De novo fatty acid synthesis during mycobacterial infection is a prerequisite for the function of highly proliferative T cells, but not for dendritic cells or macrophages. Front Immunol.

[82] Savina A, Jancic C, Hugues S, Guermonprez P, Vargas P, Moura IC (2006). NOX2 controls phagosomal ph to regulate antigen processing during crosspresentation by dendritic cells. Cell..

[83] Ding Y, Guo Z, Liu Y, Li X, Zhang Q, Xu X (2016). The lectin Siglec-G inhibits dendritic cell cross-presentation by impairing MHC class I–peptide complex formation. Nat. Immunol.

[84] Sánchez-Paulete AR, Cueto FJ, Martínez-López M, Labiano S, Morales-Kastresana A, Rodríguez-Ruiz ME (2016). Cancer immunotherapy with immunomodulatory Anti-CD137 and Anti-PD-1 monoclonal antibodies requires BATF3-dependent dendritic cells. Cancer Discov.

[85] Cheng WC, Tsui YC, Ragusa S, Koelzer VH, Mina M, Franco F (2019). Uncoupling protein 2 reprograms the tumor microenvironment to support the anti-tumor immune cycle. Nat. Immunol.

[86] Spranger S, Gajewski TF (2018). Impact of oncogenic pathways on evasion of antitumour immune responses. Nat Rev Cancer.

[87] Vander Heiden MG, DeBerardinis RJ (2017). Understanding the intersections between metabolism and cancer biology. Cell.

[88] Sullivan MR, Danai LV, Lewis CA, Chan SH, Gui DY, Kunchok T, et al. Quantification of microenvironmental metabolites in murine cancers reveals determinants of tumor nutrient availability. Elife. 2019;8:e44235. 10.7554/eLife.44235 PMC6510537 30990168

[89] Ho PC, Bihuniak JD, Macintyre AN, Staron M, Liu X, Amezquita R (2015). Phosphoenolpyruvate is a metabolic checkpoint of anti-tumor T cell responses. Cell..

[90] Reinfeld BI, Madden MZ, Wolf MM, Chytil A, Bader JE, Patterson AR (2021). Cell-programmed nutrient partitioning in the tumour microenvironment. Nature..

[91] Kelly B, O’Neill LAJ (2015). Metabolic reprogramming in macrophages and dendritic cells in innate immunity. Cell Res.

[92] Zhang Z, Liu Q, Che Y, Yuan X, Dai L, Zeng B (2010). Antigen presentation by dendritic cells in tumors is disrupted by altered metabolism that involves pyruvate kinase M2 and its interaction with SOCS3. Cancer Res.

[93] Yang M, Ma C, Liu S, Shao Q, Gao W, Song B (2010). HIF-dependent induction of adenosine receptor A2b skews human dendritic cells to a Th2-stimulating phenotype under hypoxia. Immunol Cell Biol.

[94] Noman MZ, Desantis G, Janji B, Hasmim M, Karray S, Dessen P (2014). PD-L1 is a novel direct target of HIF-1α, and its blockade under hypoxia enhanced MDSC-mediated T cell activation. J Exp Med.

[95] Li X, Wenes M, Romero P, Huang SC-C, Fendt S-M, Ho P-C (2019). Navigating metabolic pathways to enhance antitumour immunity and immunotherapy. Nat Rev Clin. Oncol.

[96] Gottfried E, Kunz-Schughart LA, Ebner S, Mueller-Klieser W, Hoves S, Andreesen R (2006). Tumor-derived lactic acid modulates dendritic cell activation and antigen expression. Blood..

[97] Brown TP, Bhattacharjee P, Ramachandran S, Sivaprakasam S, Ristic B, Sikder MOF (2020). The lactate receptor GPR81 promotes breast cancer growth via a paracrine mechanism involving antigen-presenting cells in the tumor microenvironment. Oncogene..

[98] Devalaraja S, To TKJ, Folkert IW, Natesan R, Alam MZ, Li M (2020). Tumor-derived retinoic acid regulates intratumoral monocyte differentiation to promote immune suppression. Cell..

[99] Ramakrishnan R, Tyurin VA, Veglia F, Condamine T, Amoscato A, Mohammadyani D (2014). Oxidized lipids block antigen cross-presentation by dendritic cells in cancer. J Immunol.

[100] Veglia F, Tyurin VA, Mohammadyani D, Blasi M, Duperret EK, Donthireddy L (2017). Lipid bodies containing oxidatively truncated lipids block antigen cross-presentation by dendritic cells in cancer. Nat Commun.

[101] Krengel U, Bousquet P. A molecular recognition of gangliosides and their potential for cancer immunotherapies. Front Immunol. 2014;5:325. 10.3389/fimmu.2014.00325 PMC4104838 25101077

[102] Shurin GV, Shurin MR, Bykovskaia S, Shogan J, Lotze MT, Barksdale EM (2001). Neuroblastoma-derived gangliosides inhibit dendritic cell generation and function. Cancer Res.

[103] Péguet-Navarro J, Sportouch M, Popa I, Berthier O, Schmitt D, Portoukalian J (2003). Gangliosides from human melanoma tumors impair dendritic cell differentiation from monocytes and induce their apoptosis. J Immunol.

[104] Jales A, Falahati R, Mari E, Stemmy EJ, Shen W, Southammakosane C (2011). Ganglioside-exposed dendritic cells inhibit T-cell effector function by promoting regulatory cell activity. Immunology..

[105] Sivan A, Corrales L, Hubert N, Williams JB, Aquino-Michaels K, Earley ZM (2015). Commensal bifidobacterium promotes antitumor immunity and facilitates anti-PD-L1 efficacy. Science..

[106] Routy B, Le Chatelier E, Derosa L, Duong CPMM, Alou MT, Daillère R (2018). Gut microbiome influences efficacy of PD-1-based immunotherapy against epithelial tumors. Science..

[107] Gopalakrishnan V, Spencer CN, Nezi L, Reuben A, Andrews MC, Karpinets TV (2018). Gut microbiome modulates response to anti-PD-1 immunotherapy in melanoma patients. Science..

[108] Matson V, Fessler J, Bao R, Chongsuwat T, Zha Y, Alegre M-L (2018). The commensal microbiome is associated with anti-PD-1 efficacy in metastatic melanoma patients. Science.

[109] Trompette A, Gollwitzer ES, Yadava K, Sichelstiel AK, Sprenger N, Ngom-Bru C (2014). Gut microbiota metabolism of dietary fiber influences allergic airway disease and hematopoiesis. Nat Med.

[110] Viaud S, Saccheri F, Mignot G, Yamazaki T, Daillère R, Hannani D (2013). The intestinal microbiota modulates the anticancer immune effects of cyclophosphamide. Science..

[111] Wypych TP, Pattaroni C, Perdijk O, Yap C, Trompette A, Anderson D, et al. Microbial metabolism of l-tyrosine protects against allergic airway inflammation. Nat Immunol. 2021;22:279–86. 10.1038/s41590-020-00856-3 33495652

[112] Esterházy D, Loschko J, London M, Jove V, Oliveira TY, Mucida D (2016). Classical dendritic cells are required for dietary antigen-mediated induction of peripheral T reg cells and tolerance. Nat Immunol.

[113] Ohnmacht C, Pullner A, King SBS, Drexler I, Meier S, Brocker T (2009). Constitutive ablation of dendritic cells breaks self-tolerance of CD4 T cells and results in spontaneous fatal autoimmunity. J Exp Med.

[114] Bates J, Diehl L (2014). Dendritic cells in IBD pathogenesis: an area of therapeutic opportunity?. J. Pathol.

[115] Lelouard H, Fallet M, De Bovis B, Méresse S, Gorvel J (2012). Peyer’s patch dendritic cells sample antigens by extending dendrites through M cell-specific transcellular pores. Gastroenterology..

[116] Wagner C, Bonnardel J, Da Silva C, Spinelli L, Portilla CA, Tomas J, et al. Differentiation paths of Peyer’s patch LysoDCs are linked to sampling site positioning, migration, and T cell priming. Cell Rep. 2020;31:107479. 10.1016/j.celrep.2020.03.043 32268097

[117] Rescigno M, Urbano M, Valzasina B, Francolini M, Rotta G, Bonasio R (2001). Dendritic cells express tight junction proteins and penetrate gut epithelial monolayers to sample bacteria. Nat Immunol.

[118] Niess JH, Reinecker HC (2005). Lamina propria dendritic cells in the physiology and pathology of the gastrointestinal tract. Curr Opin Gastroenterol.

[119] Sun CM, Hall JA, Blank RB, Bouladoux N, Oukka M, Mora JR (2007). Small intestine lamina propria dendritic cells promote de novo generation of Foxp3 T reg cells via retinoic acid. J Exp Med.

[120] Varol C, Vallon-Eberhard A, Elinav E, Aychek T, Shapira Y, Luche H (2009). Intestinal lamina propria dendritic cell subsets have different origin and functions. Immunity..

[121] Bogunovic M, Ginhoux F, Helft J, Shang L, Hashimoto D, Greter M (2009). Origin of the lamina propria dendritic cell network. Immunity..

[122] Atarashi K, Nishimura J, Shima T, Umesaki Y, Yamamoto M, Onoue M (2008). ATP drives lamina propria TH17 cell differentiation. Nature..

[123] Hansen IS, Krabbendam L, Bernink JH, Loayza-Puch F, Hoepel W, Van Burgsteden JA, et al. FcαRI co-stimulation converts human intestinal CD103+ dendritic cells into pro-inflammatory cells through glycolytic reprogramming. Nat Commun. 2018;9:863. 10.1038/s41467-018-03318-5 PMC5830413 29491406

[124] Torres D, Köhler A, Delbauve S, Caminschi I, Lahoud MH, Shortman K (2016). IL-12p40/IL-10 producing preCD8α/Clec9A+ dendritic cells are induced in neonates upon listeria monocytogenes infection. PLoS Pathog.

[125] Köhler A, Delbauve S, Smout J, Torres D, Flamand V Very early-life exposure to microbiota-induced TNF drives the maturation of neonatal pre-cDC1. Gut. 2020;70:511–21. 10.1136/gutjnl-2019-319700 32546472

[126] Ganal SC, Sanos SL, Kallfass C, Oberle K, Johner C, Kirschning C (2012). Priming of natural killer cells by nonmucosal mononuclear phagocytes requires instructive signals from commensal microbiota. Immunity.

[127] Baruch EN, Youngster I, Ben-Betzalel G, Ortenberg R, Lahat A, Katz L (2021). Fecal microbiota transplant promotes response in immunotherapy-refractory melanoma patients. Science..

[128] Wang H, Lu Y, Yan Y, Tian S, Zheng D, Leng D (2020). Promising treatment for type 2 diabetes: fecal microbiota transplantation reverses insulin resistance and impaired islets.. Front Cell Infect Microbiol..

[129] Davar D, Dzutsev AK, Mcculloch JA, Rodrigues RR, Pagliano O, Zidi B (2021). Fecal microbiota transplant overcomes resistance to anti – PD-1 therapy in melanoma patients. Science.

[130] Novakovic B, Habibi E, Wang SY, Arts RJW, Davar R, Megchelenbrink W (2016). β-glucan reverses the epigenetic state of LPS-induced immunological tolerance. Cell.

[131] Geva-Zatorsky N, Sefik E, Kua L, Pasman L, Tan TG, Ortiz-Lopez A (2017). Mining the human gut microbiota for immunomodulatory organisms. Cell.

[132] Swiecki M, Miller HL, Sesti-Costa R, Cella M, Gilfillan S, Colonna M (2017). Microbiota induces tonic CCL2 systemic levels that control pDC trafficking in steady state.. Mucosal Immunol..

[133] Stefan KL, Kim MV, Iwasaki A, Kasper DL (2020). Commensal microbiota modulation of natural resistance to virus infection. Cell.

[134] Schaupp L, Muth S, Rogell L, Kofoed-Branzk M, Melchior F, Lienenklaus S (2020). Microbiota-induced type i interferons instruct a poised basal state of dendritic cells. Cell..

[135] Vétizou M, Pitt JM, Daillère R, Lepage P, Waldschmitt N, Flament C (2015). Anticancer immunotherapy by CTLA-4 blockade relies on the gut microbiota. Science.

[136] Garris CS, Arlauckas SP, Kohler RH, Trefny MP, Garren S, Piot C (2018). Successful anti-PD-1 cancer immunotherapy requires T cell-dendritic cell crosstalk involving the cytokines IFN-γ and IL-12. Immunity.

[137] Mager LF, Burkhard R, Pett N, Cooke NCA, Brown K, Ramay H (2020). Microbiome-derived inosine modulates response to checkpoint inhibitor immunotherapy. Science..

[138] Tanoue T, Morita S, Plichta DR, Skelly AN, Suda W, Sugiura Y (2019). A defined commensal consortium elicits CD8 T cells and anti-cancer immunity. Nature..

[139] Nejman D, Livyatan I, Fuks G, Gavert N, Zwang Y, Geller LT (2020). The human tumor microbiome is composed of tumor type-specific intracellular bacteria. Science..

[140] Kalaora S, Nagler A, Nejman D, Alon M, Barbolin C, Barnea E (2021). Identification of bacteria-derived HLA-bound peptides in melanoma. Nature..

[141] Elinav E, Garrett WS, Trinchieri G, Wargo J (2019). The cancer microbiome. Nat. Rev. Cancer.

[142] Xavier JB, Young VB, Skufca J, Ginty F, Testerman T, Pearson AT (2020). The cancer microbiome: distinguishing direct and indirect effects requires a systemic view.. Trends Cancer..

[143] Shi Y, Zheng W, Yang K, Harris KG, Ni K, Xue L, et al. Intratumoral accumulation of gut microbiota facilitates CD47-based immunotherapy via STING signaling. J Exp Med. 2020;217:e20192282. 10.1084/jem.20192282 PMC7201921 32142585

[144] Levan SR, Stamnes KA, Lin DL, Panzer AR, Fukui E, Mccauley K (2019). Elevated faecal 12,13-diHOME concentration in neonates at high risk for asthma is produced by gut bacteria and impedes immune tolerance. Nat Microbiol..

[145] Arpaia N, Campbell C, Fan X, Dikiy S, Van Der Veeken J, Deroos P (2013). Metabolites produced by commensal bacteria promote peripheral regulatory T-cell generation. Nature..

[146] Säemann MD, Parolini O, Böhmig GA, Kelemen P, Krieger P-M, Neumüller J (2002). Bacterial metabolite interference with maturation of human monocyte-derived dendritic cells. J Leukoc Biol.

[147] Nastasi C, Candela M, Bonefeld CM, Geisler C, Hansen M, Krejsgaard T (2015). The effect of short-chain fatty acids on human monocyte-derived dendritic cells. Sci Rep.

[148] Coutzac C, Jouniaux J-MM, Paci A, Schmidt J, Mallardo D, Seck A (2020). Systemic short chain fatty acids limit antitumor effect of CTLA-4 blockade in hosts with cancer. Nat Commun..

[149] Uribe-Herranz M, Rafail S, Beghi S, Gil-De-Gómez L, Verginadis I, Bittinger K (2020). Gut microbiota modulate dendritic cell antigen presentation and radiotherapy-induced antitumor immune response. J Clin. Investig.

[150] He Y, Fu L, Li Y, He Y, Fu L, Li Y (2021). Gut microbial metabolites facilitate anticancer therapy efficacy by modulating cytotoxic CD8+ T cell immunity. Cell Metab..

[151] Ryz NR, Meddings JB, Taylor CG (2009). Long-chain inulin increases dendritic cells in the Peyer’s patches and increases ex vivo cytokine secretion in the spleen and mesenteric lymph nodes of growing female rats, independent of zinc status. Br J Nutr.

[152] Li Y, Elmén L, Segota I, Xian Y, Tinoco R, Feng Y (2020). Prebiotic-induced anti-tumor immunity attenuates tumor growth. Cell Rep.

[153] Trompette A, Gollwitzer ES, Pattaroni C, Lopez-Mejia IC, Riva E, Pernot J (2018). Dietary fiber confers protection against flu by shaping ly6c− patrolling monocyte hematopoiesis and CD8+ T cell metabolism. Immunity..

[154] Schulthess J, Pandey S, Capitani M, Rue-Albrecht KC, Arnold I, Franchini F (2019). The short chain fatty acid butyrate imprints an antimicrobial program in macrophages. Immunity..

[155] Donohoe DR, Collins LB, Wali A, Bigler R, Sun W, Bultman SJ (2012). The Warburg effect dictates the mechanism of butyrate-mediated histone acetylation and cell proliferation. Mol Cell.

[156] Campbell C, McKenney PT, Konstantinovsky D, Isaeva OI, Schizas M, Verter J (2020). Bacterial metabolism of bile acids promotes generation of peripheral regulatory T cells. Nature..

[157] Hang S, Paik D, Yao L, Kim E, Trinath J, Lu J (2019). Bile acid metabolites control TH17 and Treg cell differentiation. Nature..

[158] Song X, Sun X, Oh SF, Wu M, Zhang Y, Zheng W (2020). Microbial bile acid metabolites modulate gut ROR gamma(+) regulatory T cell homeostasis. Nature..

[159] Fiorucci S, Baldoni M, Ricci P, Zampella A, Distrutti E, Biagioli M (2020). Bile acid-activated receptors and the regulation of macrophages function in metabolic disorders. Curr. Opin. Pharmacol.

[160] Penna G, Amuchastegui S, Laverny G, Adorini L. Vitamin D receptor agonists in the treatment of autoimmune diseases: Selective targeting of myeloid but not plasmacytoid dendritic cells. J Bone Miner Res. 2007;22:V89–73. 10.1359/jbmr.07s217 18290726

[161] Gadaleta RM, Van Erpecum KJ, Oldenburg B, Willemsen ECL, Renooij W, Murzilli S (2011). Farnesoid X receptor activation inhibits inflammation and preserves the intestinal barrier in inflammatory bowel disease. Gut..

[162] Massafra V, Ijssennagger N, Plantinga M, Milona A, Ramos Pittol JM, Boes M (2016). Splenic dendritic cell involvement in FXR-mediated amelioration of DSS colitis. Biochimica Biophys Acta—Mol Basis Dis.

[163] Winkler ES, Shrihari S, Hykes BL, Handley SA, Andhey PS, Huang Y-JS, et al. The intestinal microbiome restricts alphavirus infection and dissemination through a bile acid-type I IFN signaling axis. Cell. 2020;182:901–918.e18. 10.1016/j.cell.2020.06.029 PMC7483520 32668198

[164] Hegde S, Krisnawan VE, Herzog BH, Zuo C, Breden MA, Knolhoff BL (2020). Dendritic cell paucity leads to dysfunctional immune surveillance in pancreatic cancer. Cancer Cell.

[165] Alaniz RC, Sandall S, Thomas EK, Wilson CB (2004). Increased dendritic cell numbers impair protective immunity to intracellular bacteria despite augmenting antigen-specific CD8 + T lymphocyte responses. J Immunol.

[166] Morante-Palacios O, Fondelli F, Ballestar E, Martínez-Cáceres EM (2021). Tolerogenic dendritic cells in autoimmunity and inflammatory diseases. Trends Immunol.

[167] Thwe PM, Pelgrom L, Cooper R, Beauchamp S, Reisz JA, D’Alessandro A (2017). Cell-intrinsic glycogen metabolism supports early glycolytic reprogramming required for dendritic cell immune responses. Cell Metab.

